# Analgesic efficacy and safety of nalbuphine versus morphine for perioperative tumor ablation: a randomized, controlled, multicenter trial

**DOI:** 10.1186/s13063-022-06825-5

**Published:** 2022-10-22

**Authors:** Youhua Xue, Zhengli Huang, Bingwei Cheng, Jie Sun, Haidong Zhu, Yuting Tang, Xiaoyan Wang

**Affiliations:** 1 Center of Interventional Radiology and Vascular Surgery, Department of Radiology, Zhongda Hospital, School of Medicine, Southeast University, Nanjing, 210009 Jiangsu China; 2 Department of Epidemiology and Biostatistics, School of Public Health, Southeast University, Nanjing, 210009 China; 3 Department of Anaesthesiology, Zhongda Hospital, School of Medicine, Southeast University, Nanjing, 210009 China; 4 Nursing Department, Zhongda Hospital, Medical School, Southeast University, Nanjing, 210009 China

**Keywords:** Tumor ablation, Perioperative, Pain, Nalbuphine

## Abstract

**Background:**

The study will compare the efficacy and safety of nalbuphine hydrochloride injection and morphine hydrochloride injection for perioperative analgesia in tumor ablation and the differences between the two groups regarding duration of surgery, average daily dose, patient satisfaction with analgesia, quality of life, and other indicators. Furthermore, it will evaluate the clinical application of nalbuphine and morphine for perioperative analgesia in ablation surgery and provides important reference and guidance for clinical practice.

**Methods:**

This is a randomized controlled study. Patients who were diagnosed by clinicians and required tumor ablation are enrolled and randomized to the experimental groups. In the test group, nalbuphine 80 mg + 0.9% normal saline (72 ml) is set in the patient-controlled analgesia pump, which is connected 15 min before ablation under electrocardiogram monitoring and surgery is performed immediately. The doses are as follows: initial,: 0.15 ml/kg,; background:, 0.5 ml/h,; compression:, 2 ml,; and lockout time:, 15 min. If the numeric rating scale is ≥ 4 points, the drug is administered by compression. The control group receives similar treatment under similar conditions as the test group except morphine (80 mg) is administered instead of nalbuphine (80 mg). The primary endpoints are the effective rate of analgesia and the incidence of adverse reactions (nausea and vomiting, dizziness, itching, constipation, hypoxemia, and urinary retention); the secondary endpoints are pain intensity, satisfaction with analgesia, duration of surgery, postoperative hospital stay, average daily dose, uninterrupted completion rate of surgery without complaints of pain, quality of life assessment, and vital signs.

**Discussion:**

This study, to the best of our knowledge, is the first randomized controlled trial of nalbuphine patient-controlled analgesia in ablation surgery.

**Trial registration:**

U.S. Clinical Trials Network Registration No.: NCT05073744. Registered on 11 October, 2021.

**Supplementary Information:**

The online version contains supplementary material available at 10.1186/s13063-022-06825-5.

## Administrative information

Note: the numbers in curly brackets in this protocol refer to SPIRIT checklist item numbers. The order of the items has been modified to group similar items (see http://www.equator-network.org/reporting-guidelines/spirit-2013-statement-defining-standard-protocol-items-for-clinical-trials/).

**Table:** 

Title {1}	Analgesic efficacy and safety of nalbuphine versus morphine for perioperative tumor ablation: a randomized, controlled, multicenter trial
Trial registration {2a and 2b}.	U.S. Clinical Trials Network Registration No.: NCT05073744.
Protocol version {3}	06/05/2021, V1.2
Funding {4}	This study is supported by Zhongda Hospital, Southeast University, grant number: 2021010069
Author details {5a}	Youhua Xue^1^, Zhengli Huang^1^, Bingwei Cheng^2^, Jie Sun^3^, Haidong Zhu^1^, Yuting Tang^4^, Xiaoyan Wang^4*^ ^1^Center of Interventional Radiology and Vascular Surgery, Department of Radiology, Zhongda Hospital, School of Medicine, Southeast University, Nanjing, Jiangsu 210009, China ^2^Department of Epidemiology and Biostatistics, School of Public Health, Southeast University, Nanjing 210009, China ^3^Department of Anaesthesiology, Zhongda Hospital, School of Medicine, Southeast University, Nanjing 210009, China ^4^Nursing Department, Zhongda Hospital, Medical School, Southeast University, Nanjing 210009, China
Name and contact information for the trial sponsor {5b}	The study is sponsored by grant from Yichang Humanwell Pharmaceutical Project, grant number: 20210728032
Role of sponsor {5c}	The funder only provides research funding.

## Introduction

### Background and rationale {6a}

Malignant tumors have become one of the major public health problems that critically threaten the health of the Chinese population. According to the latest statistical data, malignant tumors are the leading cause of death, accounting for 23.91% of all-cause mortality in residents of China [1]. The morbidity and mortality of malignant tumors have shown a constant rise in the past decade [2]. The World Health Organization/International Agency for Research on Cancer (WHO/IARC) released the 2020 Global Cancer Report [3], which states that there were 18.1 million new cancer cases and approximately 9.55 million deaths worldwide in 2018. The latest statistical report by the China National Cancer Center [4] declared that there were 3.929 million new cancer cases and 2.338 million cancer deaths in 2015. The most frequent cancer types included lung, gastric, colorectal, liver, breast, esophageal, and gastrointestinal cancer [5]. In recent years, biomedical discipline has continuously evolved to develop new technologies and methods, and interventional tumor therapy has grown into a comprehensive treatment option for tumor patients owing to its high efficiency, safety, minimally invasive nature, and targetability [6].

Tumor ablation is a common non-vascular interventional tumor therapy and has been recommended by several guidelines for treating diverse solid tumors, for example, liver cancer and lung cancer [7–14]. The guidelines for the treatment of liver cancer recommend radiofrequency, cryotherapy, absolute alcohol injection, and microwave ablation (MWA) as local therapies [8]. These tumor ablation techniques are guided by imaging equipment (such as CT and ultrasound) and puncture needles into the tumor. Acting on the tumor tissue by physical or chemical means makes the tumor tissue necrotic and inactive, so as to achieve the purpose of controlling and eliminating the tumor. In 2018, the number of MWA procedures performed in China has increased to 100,000, accounting for 48% of the global number of these procedures. With continuous progress and development of ablation technology as well as the gradual increase of clinical high-grade evidence, tumor ablation is expected to become one of the preferred methods for treating early-stage solid tumors and an alternative for surgery [15].

With the popularization of interventional therapy for cancer, pain caused by the therapy has become the major problem that medical staff and patients need to consider. The degree and duration of pain vary according to interventional radiology technique; additionally, there are great individual differences, such as radiofrequency ablation being accompanied by burning and tingling sensations in the viscera and skin [16]. A study on ablation therapy in patients with hepatocellular carcinoma [17] indicated that 70.6% and 25% of patients had an intraoperative pain score > 4 and > 8 points, respectively, with a higher pain level in patients with multiple ablations than in those with one ablation (6.76 ± 1.96 versus 5.28 ± 2.48). The sedation/analgesia guidelines for interventional diagnosis and treatment published by the European Society of Anesthesiology [18], the American Society of Anesthesiologists [19], the American Association of Interventional Pain Physicians [20], and the European Society of Cardiovascular and Interventional Radiology [21] recommend opioids such as morphine for analgesia before, during and/or after interventional surgery, to ensure good patient experience.

Opioids are the drugs of choice for the clinical treatment of pain, and morphine, being a classic opioid analgesic, is widely used in clinical practice [15, 22, 23]. Morphine is an opioid receptor agonist, has low lipid solubility and strong analgesic efficacy, and is mainly bio-transformed in the liver with a half-life of 2–4 h [24]. Although morphine has a positive analgesic effect, many patients cannot tolerate it and switch to other opioids due to adverse effects such as respiratory and circulatory depression [25–27], nausea and vomiting [28], and addiction [29].

Nalbuphine is a morphinan semisynthetic agonist-antagonist opioid, which exerts pharmacological effects mainly by activating kappa (κ)-receptors and antagonizing mu (μ)-receptors. It has a strong analgesic effect, and can antagonize nausea and vomiting caused by some μ receptors [30]. With intravenous administration, the onset of action is 2–3 min with a peak at 30 min, half-life of approximately 5 h, and duration of action of approximately 3–6 h. The analgesic potency is similar to that of morphine, but it has fewer side effects and higher safety. Compared with morphine, incidence of skin itching, nausea, vomiting, and respiratory depression were significantly reduced [31]. Nalbuphine is mainly used for the treatment of conditions accompanying moderate to severe pain [32], including burns, multiple trauma, orthopedic injuries, and gynecological and intra-abdominal diseases [33, 34]; it can be infused intravenously, epidurally, and subcutaneously through mechanical or microcomputer analgesic pumps.

Patient-controlled analgesia (PCA) is a pain management technique in which medical staff pre-sets the dose of analgesic drugs according to the patient's pain level and physical condition, which enables patient self-management. It is clinically applied to various types of pain, such as postoperative pain, pain due to labor, cancer, burn, and trauma, and acute pain [35–38]. Compared with traditional intravenous and subcutaneous analgesics, PCA has certain advantages: (1) lower peak concentration of the analgesic drug during analgesic treatment, minor fluctuation of plasma concentration, low incidence of respiratory depression, and reduced side effects of excessive sedation; (2) better pain control; (3) it can overcome the individual differences related to the kinetics and pharmacodynamics of analgesics through on-demand administration; (4) reduced patient wait time for medical staff to assist with pain; (5) reduced incidence of postoperative complications; (6) improved satisfaction rate of patients and their families with medical quality; and (7) reduced workload on medical staff [39].

Although relevant guideline consensus and clinical studies recommend the use of opioids for analgesia treatment in interventional surgery through intravenous, subcutaneous, and intramuscular injections, PCA, and other means, no study has evaluated the use of nalbuphine PCA for analgesia in ablation surgery so far; additionally, its efficacy and safety have not yet been demonstrated in a large number of clinical trials in China.

Therefore, a randomized controlled study was planned to compare the efficacy and safety of nalbuphine patient-controlled intravenous analgesia (PCIA) with morphine PCIA for perioperative analgesia in ablation surgery including patients with different types of cancer and ablation surgery, surgical conditions, and analgesic methods in hospitals at all levels to provide a reference and suggestions for clinically important standardized perioperative analgesia in ablation surgery.

### Objectives {7}

The purpose of this study is to compare the efficacy and safety of nalbuphine hydrochloride injection and morphine hydrochloride injection for perioperative analgesia in tumor ablation, evaluate clinically applicable nalbuphine and morphine in analgesic regimen for perioperative ablation, and provide an important reference for the development of clinical practice guidelines.

### Trial design {8}

This study is a superiority trial. We designed it by a multicenter, single-blind, randomized, parallel, positive-controlled clinical study to observe the efficacy and safety of nalbuphine hydrochloride injection and morphine hydrochloride injection for perioperative analgesia in tumor ablation. With such a study design, we hope to find a more effective and safe method of analgesia in the perioperative period for tumor ablation.

## Methods: participants, interventions, and outcomes

### Study setting {9}

The setting of this study is the interventional ward and interventional catheterization operating room of the hospital.

### Eligibility criteria {10}

Patients undergoing tumor ablation procedures are included.

Participants who meet the following inclusion criteria are eligible for this study:

- Patients who are diagnosed by clinicians and require tumor ablation surgery;
- Patients who provide written informed consent; and
- Patients aged 18–80 years.

The exclusion criteria are as follows:

1. Allergy to contrast agents, test drugs, or other ingredients in the drug formulation;
2. History of drug abuse, chronic pain, and mental illness;
3. Use of monoamine oxidase inhibitors within 14 days before randomization;
4. Pregnancy, lactation, and intolerance of surgery for other reasons;
5. Difficulty correctly expressing needs or preferences;
6. Poor compliance, inability to complete the trial according to the study protocol;
7. Participation in other drug trials within 30 days before inclusion; and
8. Not eligible to participate in this trial based on investigator opinion.

### Who will take informed consent? {26a}

Researchers will obtain informed consent or assent from potential trial participants or authorized surrogates. Informed consent was given verbally and in writing, dated and signed by the subject.

### Additional consent provisions for collection and use of participant data and biological specimens {26b}

Not applicable. We have described the terms of collection and use of participant data and biospecimens in the informed consent.

## Interventions

### Explanation for the choice of comparators {6b}

Opioids are the drugs of choice for the clinical treatment of pain, and morphine, as a classical opioid, is widely used in clinical practice.

### Intervention description {11a}

#### Intervention group

For the intervention group, nalbuphine 80 mg + 0.9% normal saline (72 ml) is pre-set in the PCA pump, and the pump is connected 15 min before ablation. Surgery is performed immediately under electrocardiogram (ECG) monitoring, with an initial dose of 0.15 ml/kg, a background dose of 0.5 ml/h, a compression dose of 2 ml, and a lockout time of 15 min. If the Numeric Rating Scale (NRS) score is ≥ 4 points, the drug is administered by compression. Pain intensity, incidence of adverse reactions (nausea and vomiting, dizziness, pruritus, constipation, hypoxemia, and urinary retention), satisfaction with analgesia, duration of surgery, postoperative hospital stay, average daily dose, uninterrupted completion rate of the surgery without complaints of pain, quality of life assessment, and vital signs are evaluated every 1 min and 5 min after ablation at the end of surgery, and 2 h, 6 h, 12 h, 24 h, and 48 h after surgery.

#### Control group

For the control group, morphine 80 mg + 0.9% normal saline (72 ml) is added in the PCA pump, which is connected 15 min before ablation. Surgery is performed immediately under ECG monitoring. The initial dose is 0.15 ml/kg, background dose is 0.5 ml/h, compression dose is 2 ml, and lockout time is 15 min. If the NRS is ≥ 4 points, the drug is administered by compression. Pain intensity and the incidence of adverse reactions (nausea and vomiting, dizziness, pruritus, constipation, hypoxemia, and urinary retention) were evaluated every 1 min and 5 min after ablation at the end of the surgery, and 2 h, 6 h, 12 h, 24 h, and 48 h after surgery. Satisfaction with analgesia, duration of surgery, postoperative hospital stay, average daily dose, uninterrupted completion rate of surgery without complaints of pain, quality of life assessment, and vital signs are also assessed.

### Criteria for discontinuing or modifying allocated interventions {11b}

During the study, the study can be terminated early/closed due to the following reasons: (1) Serious adverse events related to the test drug occurred during the study; (2) The effect of the drug was found to be too poor in the test, and there was no need to continue the study; (3) Major mistakes in the clinical trial protocol were found in the trial, and it was difficult to evaluate the drug effect; (4) The drug regulatory authority or the ethics committee requested to terminate the approved research.

An individual participant’s disenrollment criteria are as follows: (1) inability to complete the observation due to adverse events, (2) withdrawal invited by the investigator (poor compliance, serious adverse events), and (3) combination of prohibited drugs during the study period that prevented satisfaction and effectiveness evaluation.

### Strategies to improve adherence to interventions {11c}

To improve subject compliance, open recruitment was used, and patients were screened according to the inclusion and exclusion criteria of the study; informed consent should follow the Standard Operating Procedures for Signing Informed Consent, and after the investigator fully informed the subjects about the trial, the subjects’ right to decide whether or not to participate in the trial was respected either voluntarily or after discussion with their families; follow-up visits were arranged for the subjects during the trial, and all examinations were completed in strict accordance with the protocol.

### Relevant concomitant care permitted or prohibited during the trial {11d}

During the study period,in addition to the test drug, other analgesic drugs (such as non-steroidal analgesics, antidepressants, etc.) shall not be newly added during the test period, but other drugs that do not affect the efficacy of opioids can be used.

### Provisions for post-trial care {30}

The investigator will make every effort to prevent and treat any harm that may result from this study. If any damage related to the study occurs in the study, the treatment cost and corresponding economic compensation will be provided in accordance with the provisions of The Quality Management Standard for Drug Clinical Trials in China.

### Outcomes {12}

#### Main study outcome

Analgesic effective rate: Analgesic effective rate = number of patients with effective analgesia/total number of cases × 100%. NRS score (at rest) ≤ 3 points at all assessment time points is considered as effective analgesia and assessed at 1 min and 5 min after ablation, at the end of the surgery, and at 2 h, 6 h, 12 h, 24 h, and 48 h after surgery. The NRS is used for assessment, which consists of 11 numbers with the same interval from 0 to 10, with 0 representing “no pain” and 10 representing “most intense pain.” Patients are asked to choose one number to represent the pain intensity score.

#### Secondary outcomes

Pain intensity: Pain intensity is assessed using NRS at 1 min and 5 min after the start of ablation, at the end of the surgery, and at 2 h, 6 h, 12 h, 24 h, and 48 h after surgery (at rest, during coughing, or during activity).

Evaluation of the degree of analgesic satisfaction: (A) before leaving the operating room to assess the patient's satisfaction with the analgesic effect; (B) before leaving the operating room, the surgeon and surgical nurses implement the satisfaction assessment. The satisfaction score uses the visual analog scale (VAS), with a Vernier ruler of 10 cm in length, marked with 10 scales, 0 point and 10 points at both ends, respectively. The patient, surgeon, and surgical nurses mark the corresponding position representing their comfort on the ruler, and the follower score it according to the position marked by the patient. A score of 0 represents extreme dissatisfaction and a score of 10 represents extreme satisfaction.

Duration of surgery: Time from the start to the end of ablation is calculated in minutes.

Postoperative hospital stay: Time from the end of surgery to discharge is calculated in days.

Average daily dose: The ratio of total dose to days of medication is calculated in milligrams.

Uninterrupted completion rate of surgery without complaints of pain: The rate is calculated as follows:

$$\mathrm{Number}\;\mathrm{of}\;\mathrm{patients}\;\mathrm{with}\;\mathrm{uninterrupted}\;\mathrm{completion}\;\mathrm{of}\;\mathrm{surgery}\;\mathrm{and}\;\mathrm{without}\;\mathrm{complaints}\;\mathrm{of}\;\mathrm{pain}/\mathrm{total}\;\mathrm{number}\;\mathrm{of}\;\mathrm{cases}\;\times\;100\%$$

Quality of life assessment: One week after surgery, quality of life is assessed through a telephone interview (using EORTC Quality of Life Inventory QLQ-C30 (V3.0), Additional file 1).

### Participant timeline {13}

Time schedule of enrolment, interventions, assessments, and visits for participants, see Additional file 2.

### Sample size {14}

The sample size and case allocation included patients who underwent tumor ablation. NRS score (at rest) ≤ 3 points during operation and within 48 h after the operation was defined as effective. Assuming that the effective rate was 90% in the nalbuphine group and 90% in the morphine group, the clinical cut-off value for non-inferiority between the two groups was 10% if the nalbuphine group is non-inferior to the morphine group, setting one-sided α-error level = 0.025, *β* = 0.2 (power = 0.8), and the sample size ratio of the two groups 1:1. Overall, 142 patients were required for each group calculated using the PASS15.0 software, and a total of 284 patients were required for the two groups. Based on the dropout rate of 10% (284 ÷ 0.9 = 316), a total of approximately 316 patients were planned to be included.

### Recruitment {15}

The study was performed based on open recruitment, and patients were screened according to the inclusion and exclusion criteria of the study.

## Assignment of interventions: allocation

### Sequence generation {16a}

The subjects are randomly assigned to the experimental group and the control group by computer-generated random numbers.

### Concealment mechanism {16b}

Only the patient’s random number and initials may appear on the electronic case report form. Random numbers are generated according to the interactive web response system.

### Implementation {16c}

Electronic Data Capture System will generate the allocation sequence, hospitals participating in the study will enroll participants, and research doctors in the hospital will assign participants to interventions.

## Assignment of interventions: blinding

### Who will be blinded {17a}

Among all trial participants and personnel, only patients who are enrolled will be blinded. They did not know what medications they were using.

### Procedure for unblinding if needed {17b}

When a serious adverse event occurs, the investigator decides whether to unblind.

## Data collection and management

### Plans for assessment and collection of outcomes {18a}

#### Main study outcomes

The NRS was used for assessment, and the NRS consisted of 11 numbers with the same interval from 0 to 10, with 0 representing “no pain” and 10 representing “most intense pain.” Patients chose one number to represent the pain intensity at their score. NRS score (at rest) ≤ 3 points at all assessment time points was considered as effective analgesia and was assessed at 1 min, 5 min, at the end of surgery, and at 2 h, 6 h, 12 h, 24 h, and 48 h after the start of ablation.

#### Secondary outcomes

Pain intensity: Pain intensity was assessed by NRS at 1 min and 5 min after the start of ablation, at the end of surgery, and at 2 h, 6 h, 12 h, 24 h, and 48 h after surgery (at rest, during coughing, or during activity). Analgesic satisfaction: Evaluation of the degree of analgesic satisfaction. (A) Before leaving the operating room to assess the patient’s satisfaction with the analgesic effect; (B) before leaving the operating room, the surgeon and surgical nurse implement the satisfaction assessment; the satisfaction score uses the VAS, with a vernier ruler of 10 cm in length, marked with 10 scales, 0 point and 10 points at both ends, respectively, so that the patient, surgeon, and surgical nurse can mark the corresponding position that can represent their comfort on the ruler, and the follower can score it according to the position marked by the patient. A score of 0 represents extreme dissatisfaction and a score of 10 represents extreme satisfaction. Quality of life: One week after surgery, quality of life was assessed by telephone (using EORTC Quality of Life Inventory QLQ-C30 (V3.0).

We also collected patient demographics, history, physical and laboratory examinations, and surgical information, including tumor type, number of tumors ablated, size and location of lesions, and ablation technique.

All of these informations were recorded in the Case Report Form.

### Plans to promote participant retention and complete follow-up {18b}

We will manage the subjects. (1) Patients were screened according to the inclusion and exclusion criteria of the study using an open recruitment approach. (2) The investigator collects personal data such as the subject’s name, contact information, home address, disease history and pain history in a timely manner. (3) The informed consent should follow the Standard Operating Procedures for Signing Informed Consent, and the investigator respects the subject's right to decide whether or not to participate in the trial, either voluntarily or after consultation with family members, after fully informing the subject about the trial. (4) Arrange follow-up visits for subjects during the trial and complete all examinations in strict accordance with the protocol. (5) Educate subjects not to use drugs prohibited in the trial on their own and to inform the investigator of information related to the combined use of drugs (e.g., name of drug, dose, duration of use, reason for use) in a timely manner at the time of follow-up. (6) Educate the subject that other tests and treatments are required due to the combination of other diseases, and inform the investigator promptly or at the next follow-up visit, and the investigator should record the relevant information promptly after learning it. (7) Inform subjects of possible adverse reactions during the study, and if any physical discomfort occurs during the course of the trial, inform the investigator in a timely manner so that the investigator can decide on a treatment plan. (8) Inform the subjects that their information during the study period will be kept confidential by the investigator and that the subjects are required not to freely divulge the trial study information to the public.

### Data management {19}

An electronic data capture system is used for data collection in this study. According to the GCP requirements for clinical trials in China, the data shall be maintained for 5 years after the termination of the clinical trial.

1. The full analysis set (FAS) of the analysis dataset includes all subjects who were randomized, received the study drug, and had at least one efficacy evaluation data after medication. In case of missing data of the primary efficacy endpoint, the last observation carried forward is applied according to the intention-to-treat principle. The missing values for the comparability analysis and secondary efficacy measures are not carried forward and were based on the analysis dataset obtained in the FAS.
2. Per protocol set (PPS) is a dataset generated from subjects who are fully compliant with the trial protocol; compliance includes receiving treatment, availability of the primary endpoint measures, and no major trial protocol violations. The per-protocol analysis is used mainly for the primary efficacy measures.
3. Safety set (SS) refers to all subjects who are randomized, received the study drug, and have at least one post-baseline safety assessment.

#### Statistical analysis method

1. The number of included and completed cases in the overall and each site is listed in the case analysis, and three analysis datasets (FAS, PPS, and SS) are generated. Dropouts and excluded cases along with the reasons for their dropping out and exclusion are listed.
2. Demographic and baseline data analysis: Descriptive statistics of demographic data and other baseline characteristic values: For continuous variables, total number of cases, mean, standard deviation, median, and minimum and maximum values are calculated. The t-test will be used for normally-distributed data and the rank-sum test will be used for data with skewed distribution. Frequency and proportion are calculated for countable and grade data. The chi-square test will be used for disordered classification data, and the rank-sum test will be used for ordinal data. Inferential statistical results (*P*-values) are presented based on descriptive statistics.
3. Efficacy analysis:
  A. Analysis of the primary efficacy indicators (overall response rate): The overall response rate is compared using the chi-square test, corrected chi-square test, or exact probability test. The 95% confidence interval of the difference in response rates between the two groups will be calculated, and if the lower limit of the confidence interval is greater than −10%, the result will be considered non-inferior.
  B. Analysis of the secondary efficacy indicators: Differences in the scores of NRS at each time point and the total score between the two groups are compared by the *t*-test or rank-sum test; differences in the physician satisfaction VAS score and the nurse satisfaction VAS score between the groups are compared by the t-test or rank-sum test; the difference between the two groups regarding willingness to undergo ablation surgery another time will be compared by the chi-square test, corrected chi-square test, or exact probability test; differences between the two groups with respect to duration of surgery, postoperative hospital stay, and average daily dose will be compared by the t-test or rank-sum test; the difference in the uninterrupted completion rate of surgery without complaints of pain between the groups will be compared by the chi-square test, corrected chi-square test, or exact probability test.
4. Safety analysis: The incidence of adverse reactions will be calculated, their frequency and proportion were systematically listed, and the percentage calculated. A detailed list of cases of various adverse events (AEs), the number and rate of cases, conversion of physical examination indicators, ECG, respiratory and circulatory indicators, and laboratory indicators that “turn normal to abnormal” or “intensify abnormal” after the test, and list clinical explanations will be recorded.

### Confidentiality {27}

The medical records and information of participants will be kept completely at the hospital. Researchers, ethics committees, drug regulatory authorities, and health commission regulatory authorities will be allowed access to participants’ medical records. Any public reporting of the results of this study will not disclose the individual identity of the participants. In accordance with the principles of medical research ethics, except for personal privacy information, the trial data will be available for public inquiry and sharing, which will be limited to web-based electronic databases to ensure that no personal privacy information will be disclosed.

Researchers must ensure that the privacy of clinical trial subjects is maintained. In all documents submitted to the sponsor, only the subject number of the clinical trial shall be used to identify the participants of the trial, and the name and hospitalization number of the subjects shall not be specified. The investigator must maintain the name and address of the clinical trial subject and the enrollment form corresponding to the clinical study patient number. These entry forms are kept strictly confidential by the investigator and cannot be submitted to the sponsor. An electronic data capture system was used for data collection in this study. According to the GCP requirements of China, the clinical trial data shall be kept for 5 years after the termination of the clinical trial.

### Plans for collection, laboratory evaluation, and storage of biological specimens for genetic or molecular analysis in this trial/future use {33}

Not applicable. No biological specimens for genetic or molecular analysis are collected, evaluated, or stored in this trial.

## Statistical methods

### Statistical methods for primary and secondary outcomes {20a}

All subjects who were randomized, received the trial drug, and had data on at least one post-dose efficacy evaluation were included in the outcome analysis. The number of different organ tumors, single or multiple tumor tissues, and different ablation techniques were compared between the two groups to determine whether they were comparable.

Statistical analyses are performed using the SAS 9.4 software. Unless otherwise specified, the level of statistical significance was set at *P* ≤ 0.05. For a non-inferiority test, a one-sided test will be used, and *P* ≤ 0.025 are considered statistically significant.

### Interim analyses {21b}

During the study, the study can be terminated early/the study center can be closed for the following reasons :(1) serious adverse events related to the experimental drug occur during the study; (2) the effect of the drug was found to be so poor that it was not necessary to continue the study; (3) it was found that there were significant errors in the clinical trial plan, and it was difficult to evaluate the drug effect; and (4) the pharmaceutical supervisory and administrative department or approved by the ethics commission has demanded an end to study early termination/close study center may be temporary, may also be permanent once decided to terminate research/close research center, the sponsor shall properly handle the relevant matters after the termination of research/close research center, and timely notify the research unit Good Clinical Practice. The archived research records shall be retained for future reference, and other relevant research materials (including partially completed blank research materials and investigatory drugs) shall be handed over to the sponsor according to the relevant requirements in the GCP.

### Methods for additional analyses (e.g., subgroup analyses) {20b}

Analysis of secondary efficacy indicators: the difference between the scores of NRS at each time point and the total score is compared by *t*-test or rank sum test; the difference between the physician satisfaction VAS score and the nurse satisfaction VAS score is compared by *t*-test or rank sum test; the difference between the rates of willing to undergo ablation surgery again is compared by chi-square test or corrected chi-square test or exact probability method; the difference between the duration of surgery is compared by *t*-test or rank sum test; the difference between the postoperative hospital stay is compared by *t*-test or rank sum test; the difference between the daily dosage is compared by *t*-test or rank sum test; the difference between the rates of uninterrupted completion of surgery without complaints of pain is compared by chi-square test or corrected chi-square test or exact probability method. Safety analysis: calculate the incidence of adverse reactions; systematically list the frequency and frequency of adverse reactions, and calculate the percentage; a detailed list of cases of various adverse events; a detailed list of cases of various adverse reactions; the number and rate of cases and conversion of physical examination indicators, ECG, respiratory and circulatory indicators, and laboratory indicators that “turn normal to abnormal” or “intensify abnormal” after the test; and list physical examination indicators, ECG, respiratory and circulatory indicators, and abnormal cases of laboratory indicators and clinical explanations.

### Methods in analysis to handle protocol non-adherence and any statistical methods to handle missing data {20c}

Not applicable. Non-compliance with the protocol and missing data, we plan not to use as part of the statistical analysis.

### Plans to give access to the full protocol, participant-level data and statistical code {31c}

The data shall not be used without the permission of the principal investigator in the responsible unit.

The principal investigator of the research unit will have full access to the final data so that the results can be properly analyzed and reported academically.

## Oversight and monitoring

### Composition of the coordinating center and trial steering committee {5d}

The group authorized a GCP-qualified clinical inspector to conduct surveillance.

### Composition of the data monitoring committee, its role, and reporting structure {21a}

The clinical study is subject to a quality assurance audit by the sponsor or a person authorized by the sponsor. GCP reviews may also be conducted by the drug approval department. Quality auditors and inspectors have access to all medical records, study-related documents and correspondence, and informed consent documents for the clinical trial.

### Adverse event reporting and harms {22}

#### Primary safety indicators

Incidence of common adverse reactions to opioid drugs, such as nausea, vomiting, vertigo, constipation, itching, urinary retention, hypoxemia, respiratory depression, drowsiness, delirium, and myoclonus are recorded. AEs are graded 1–5 according to the Common Terminology Criteria for Adverse Events 5.0 (CTCAE 5.0) of the National Cancer Institute (NCI).

- Grade 1: mild: asymptomatic or mild, clinical or diagnostic findings, no treatment required;
- Grade 2: moderate: minimal, local, or non-invasive treatment required, limiting age-appropriate instrumental activities of daily living;
- Grade 3: serious or medically significant but not immediately life-threatening, hospitalization or prolongation of hospitalization indicated, disabling, limiting self-care activities of daily living;
- Grade 4: life-threatening, urgent treatment required; and
- Grade 5: death related to AE.

If an AE is not listed in the NCI toxicity grading scale, it can be judged according to the following criteria:

- Grade I (mild): uncomfortable feeling, but does not affect normal daily activities;
- Grade II (moderate): uncomfortable degree enough to reduce or affect normal daily activities;
- Grade III (severe): unable to work or do normal daily activities; and
- Grade IV (fatal): disabling or fatal.

#### Secondary safety indicators

Physical examination: body temperature, respiration, pulse, heart rate, and blood pressure will be routinely measured; ECG and respiratory and circulatory parameters will be monitored during treatment;

Laboratory tests: hematology, biochemistry, and urinalysis.

#### Safety evaluation

##### Observation of AEs

Hypotension (0/1/2): Grade 0: systolic blood pressure (SBP) > 70% or 80/50 mm Hg before surgery; Grade 1: SBP < 70% or 80/50 mm Hg before surgery; Grade 2: SBP < 60% or 70/40 mm Hg before surgery. The patients who develop hypotension are treated with ephedrine.

Heart rate and heart rhythm changes (0/1): 1: heart rate less than 50 beats/min, more than 120 beats/min, or occurrence of arrhythmia; 0: otherwise. Atropine was given for the above conditions.

Hypoxemia (1/2/3/4): Grade 0: 96–100%; Grade 1: 91–95%; Grade 2: 86–90%; Grade 3: < 85%. If Hypoxemia occurred, jaw support was given, and if pulse oxygen saturation was < 90%, oxygen inhalation through a respiratory sac mask was provided.

Upper airway obstruction (0/1/2): Grade 0: no upper airway obstruction; Grade 1: mild snoring but normal oxygen saturation (SpO_2_) maintained; Grade 2: snoring, which must be relieved by oropharyngeal airway or jaw support, or normal SpO_2_ could not be maintained.

Respiratory depression/apnoea (0/1/2): Grade 0: respiratory rate > 12 times/min; Grade 1: 6 times/min < respiratory rate < 12 times/min; Grade 2: respiratory rate < 6 times/min.

Body movement (including retching and swallowing, limb movement) (0/1/2): 0: no body movement; 1: general body movement (toe movement, manual, not affecting the body movement examined); 2: severe body movement (leg movement or hip movement, affecting the body movement examined).

In the Case Report Form, the “Adverse Event Record Form” is maintained, and the investigators record in detail any AEs that occur in the case, particularly those related to analgesic medication. These must be recorded in the original data with medical terms and copied to the Case Report Form. The records of AEs shall include a description of AEs and all related symptoms, occurrence time, severity, duration, measures taken, final result, and outcome.

### Frequency and plans for auditing trial conduct {23}

All data obtained during clinical studies must be handled appropriately to ensure the rights and privacy of patients participating in clinical studies. The investigator must consent to the inspectors/auditors/inspectors to access and review the clinical study data as required to verify the accuracy of the original data and to understand the progress of the study. If it is not possible to verify the original records, the investigator should agree to assist the inspector/auditor/inspector in further verifying the quality control of the data.

### Plans for communicating important protocol amendments to relevant parties (e.g. trial participants, ethical committees) {25}

Prior to the study inception, the study protocol was submitted to and approved by the Ethics Committee (approved on July 8, 2021). We are in the process of conducting this study. The first case enrollment was on October 11, 2021. All protocol modifications were determined after the final version of the study protocol was approved in writing by the Ethics Committee.

### Dissemination plans {31a}

The findings will be disseminated through academic presentations at national and international conferences in the interventional field, as well as through publications in journals. According to the results of the study, we will recommend the analgesic regimen for perioperative analgesia in tumor ablation to provide an important reference for the development of clinical practice guidelines.

All participating investigators fully authorize the principal investigator of the study unit responsible for the study to make the first publication/ or first publication of the results. No other publication will be permitted prior to initial publication. Any subsequent publication or publication by study participants must be approved by the principal investigator in charge of the study and cite the study and the initial publication. A final decision on any manuscript/abstract/briefing will be made by the principal investigator of the research unit responsible. All manuscripts/abstracts/briefs must be sent to the responsible research unit for internal review at least forty-five calendar days prior to submission.

## Discussion

The popularity of COVID-19 will affect the hospitalization of patients, so the enrollment rate of this study may be affected by the epidemic and may be delayed.

### Trial status

The protocol version number V1.2 and date is 06/05/2021. The planned start date for recruitment and the approximate date of completion of recruitment are from 07/09/2021 to 07/09/2023. We are in the process of conducting this study. The first case enrollment was on 10/11/2021.

## Supplementary Information

**Additional file 1.** EORTC Quality of Life Questionnaire QLQ-C30 (V3.0).

**Additional file 2.** SPIRIT schematic diagram.

## Data Availability

The principal investigator of the research unit will have full access to the final data so that the results can be properly analyzed and reported academically. The data shall not be used without the permission of the principal investigator in the responsible unit.

## Abbreviations

AEs: Adverse events
ECG: Electrocardiogram
FAS: Full analysis set
GCP: Good clinical practice
MWA: Microwave ablation
NCI: National cancer institute
NRS: Numeric rating scale
PCA: Patient-controlled analgesia
PCIA: Patient-controlled intravenous analgesia
PPS: Per protocol set
SBP: Systolic blood pressure
SS: Safety set
VAS: Visual analog scale
