# Supplementary material for: Analgesic efficacy and safety of nalbuphine versus morphine for perioperative tumor ablation: a randomized, controlled, multicenter trial
Source: Trials. 2022 Oct 22;23:887. doi: 10.1186/s13063-022-06825-5 (PMC9587534; doi:10.1186/s13063-022-06825-5)
Supplement: Supplementary file 1 — Additional file 1. EORTC Quality of Life Questionnaire QLQ-C30 (V3.0). [file 13063_2022_6825_MOESM1_ESM.docx]

**Appendix 1**

**EORTC Quality of Life Questionnaire QLQ-C30 (V3.0)**

We would like to know some situations about you and your health. Please answer all the questions in person. There is no "yes" or "no" right answer. Only circle the number that best reflects your situation. The information provided by you will remain strictly confidential.

|  |  | | | | | | | **None** | **A little** | **Quite** | **Most** |
| --- | --- | --- | --- | --- | --- | --- | --- | --- | --- | --- | --- |
| 1 | Are you having trouble doing strenuous activities like lifting heavy shopping bags or suitcases? | | | | | | | 1 | 2 | 3 | 4 |
| 2 | Are you having trouble walking long distances? | | | | | | | 1 | 2 | 3 | 4 |
| 3 | Do you have trouble walking short distances outdoors? | | | | | | | 1 | 2 | 3 | 4 |
| 4 | Do you need to stay in bed or chair during the day? | | | | | | | 1 | 2 | 3 | 4 |
| 5 | Do you need help for eating, dressing, bathing, or using toilet? | | | | | | | 1 | 2 | 3 | 4 |
| **In the past week:** | | | | | | | | **None** | **A little** | **Quite** | **Most** |
| 6 | Were you limited in your work and daily activities? | | | | | | | 1 | 2 | 3 | 4 |
| 7 | Were you limited in your hobbies or leisure activities? | | | | | | | 1 | 2 | 3 | 4 |
| 8 | Have you experienced shortness of breath? | | | | | | | 1 | 2 | 3 | 4 |
| 9 | Have you suffered pain? | | | | | | | 1 | 2 | 3 | 4 |
| 10 | Did you feel to take a break? | | | | | | | 1 | 2 | 3 | 4 |
| 11 | Have you experienced trouble sleeping? | | | | | | | 1 | 2 | 3 | 4 |
| 12 | Did you feel weak? | | | | | | | 1 | 2 | 3 | 4 |
| 13 | Have you lost appetite (had no appetite)? | | | | | | | 1 | 2 | 3 | 4 |
| 14 | Did you feel sick? | | | | | | | 1 | 2 | 3 | 4 |
| 15 | Have you vomited? | | | | | | | 1 | 2 | 3 | 4 |
| 16 | Did you suffer from constipation? | | | | | | | 1 | 2 | 3 | 4 |
| 17 | Did you have diarrhoea? | | | | | | | 1 | 2 | 3 | 4 |
| 18 | Did you observe tiredness? | | | | | | | 1 | 2 | 3 | 4 |
| 19 | Did pain interfere with your daily activities? | | | | | | | 1 | 2 | 3 | 4 |
| 20 | Did you have trouble concentrating on activities like reading a newspaper or watching TV? | | | | | | | 1 | 2 | 3 | 4 |
| 21 | Did you feel nervous? | | | | | | | 1 | 2 | 3 | 4 |
| 22 | Did you feel worried? | | | | | | | 1 | 2 | 3 | 4 |
| 23 | Did you feel irritable? | | | | | | | 1 | 2 | 3 | 4 |
| 24 | Did you feel depressed (had low mood)? | | | | | | | 1 | 2 | 3 | 4 |
| 25 | Did you have difficulty remembering? | | | | | | | 1 | 2 | 3 | 4 |
| 26 | Did your physical condition or treatment affect your family life? | | | | | | | 1 | 2 | 3 | 4 |
| 27 | Did your medical condition or treatment affect your social activities? | | | | | | | 1 | 2 | 3 | 4 |
| 28 | Did your physical condition or treatment make you financially unstable? | | | | | | | 1 | 2 | 3 | 4 |
| **For the following questions, please circle the number between 1 and 7 that best applies to you.** | | | | | | | | | | | |
| 29 | How would you rate your overall health during the past week? | | | | | | |  |  |  |  |
|  | 1 | 2 | 3 | 4 | 5 | 6 | 7 |  |  |  |  |
|  | Very poor |  | | | | | Very good |  |  |  |  |
| 30 | How would you rate your overall quality of life during the past week? | | | | | | |  |  |  |  |
|  | 1 | 2 | 3 | 4 | 5 | 6 | 7 |  |  |  |  |
|  | Very poor |  | | | | | Very good |  |  |  |  |
