# Supplementary material for: Analgesic efficacy and safety of nalbuphine versus morphine for perioperative tumor ablation: a randomized, controlled, multicenter trial
Source: Trials. 2022 Oct 22;23:887. doi: 10.1186/s13063-022-06825-5 (PMC9587534; doi:10.1186/s13063-022-06825-5)
Supplement: Supplementary file 2 — Additional file 2. SPIRIT schematic diagram. [file 13063_2022_6825_MOESM2_ESM.docx]

**Appendix 2: SPIRIT schematic diagram**

|  | | | Enrolment | Surgery | | | | | Postsurgical courses | | | | | | |
| --- | --- | --- | --- | --- | --- | --- | --- | --- | --- | --- | --- | --- | --- | --- | --- |
| Time point | | |  | Preablation | Ablation | Postablation | | Surgery end | 2 Hour | 6 Hour | 12 Hour | 24 Hour | 48 Hour | one week | out hospital |
|  |  |  |  | -15min | 0 | 1min | 5min |  |  |  |  |  |  |  |  |
| Enrolment: |  | |  |  |  |  |  |  |  |  |  |  |  |  |  |
|  | Inclusion and exclusion criteria | | × |  |  |  |  |  |  |  |  |  |  |  |  |
|  | Informed consent | | × |  |  |  |  |  |  |  |  |  |  |  |  |
|  | Demographics | | × |  |  |  |  |  |  |  |  |  |  |  |  |
|  | Randmoisation | | × |  |  |  |  |  |  |  |  |  |  |  |  |
| Interventions: |  | |  |  |  |  |  |  |  |  |  |  |  |  |  |
|  | Nalbuphine group | |  | × |  |  |  |  |  |  |  |  |  |  |  |
|  | Morphine group | |  | × |  |  |  |  |  |  |  |  |  |  |  |
| Assessments: |  | |  |  |  |  |  |  |  |  |  |  |  |  |  |
|  | Analgesic effective rate | |  |  |  |  |  |  |  |  |  |  |  |  |  |
|  | Pain intensity（NRS） | |  |  |  | × | × | × | × | × | × | × | × |  |  |
|  | Adverse reactions | |  |  |  | × | × | × | × | × | × | × | × |  |  |
|  | Analgesic satisfaction | Analgesic effect |  |  |  | × | × | × | × | × | × | × | × |  |  |
|  |  | Medical staffs |  |  |  |  |  | × |  |  |  |  |  |  |  |
|  | Duration of surgery(min) | |  |  | 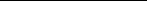♦ |  | | ♦ |  |  |  |  |  |  |  |
|  | Postoperative hospital stay(days) | |  |  |  |  |  | 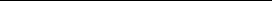♦ |  | | | | | | ♦ |
|  | Average daily dose | |  |  |  |  |  |  |  |  |  |  |  |  |  |
|  | Rate of uninterrupted completion of surgery without complaints of pain | |  |  |  | × | × | × | × | × | × | × | × |  |  |
|  | Quality of life | |  |  |  |  |  |  |  |  |  |  |  | × |  |
|  | Vital signs | |  |  |  | × | × | × | × | × | × | × | × |  |  |
